# Correction: Cost-sensitive multi-kernel ELM based on reduced expectation kernel auto-encoder

**DOI:** 10.1371/journal.pone.0339988

**Published:** 2025-12-31

**Authors:** Wang Yongqiang, Liang Yixuan

Wang Yongqiang should be included in the author byline. Wang Yongqiang should be listed as the first author, and his affiliation is: Shaanxi University of International Trade & Commerce, Xi’an, Shaanxi, 712046, P.R. China. The contributions of this author are as follows: Conceptualization, Data Curation, Formal Analysis, Investigation, Validation, Visualization and Writing – Review & Editing

The correct citation is: Yongqiang, W, Yixuan L (2025) Cost-sensitive multi-kernel ELM based on reduced expectation kernel auto-encoder. PLoS ONE 20(2): e0314851. https://doi.org/10.1371/journal.pone.0314851
