# Supplementary material for: Cost-sensitive multi-kernel ELM based on reduced expectation kernel auto-encoder
Source: PLoS One. 2025 Feb 13;20(2):e0314851. doi: 10.1371/journal.pone.0314851 (PMC11824986; doi:10.1371/journal.pone.0314851)
Supplement: Attachment — Submitted filename: Response to Reviewers.docx [file pone.0314851.s001.docx]

Dear Editors:

We would like to express our sincere appreciation for your letter and comments concerning our manuscript entitled ‘Cost-sensitive Multi-kernel ELM based on Reduced Expectation Kernel Auto-encoder’.

These comments are all valuable and very helpful for revising and improving our paper, as well as the important guiding significance to our researches. We have revised the paper carefully according to these comments and the main corrections and responds are described below. We would like to re-submit it for your consideration. We hope that the revision is acceptable, and we look forward to hearing from you soon.

With best wishes

Yours sincerely

Liang Yixuan

# Responses to the comments:

**(The modified contents are marked in red.)**

Excepted for the modifications listed below, A thorough copy editing is performed. Please refer to the revised manuscript for more details.

## ACADEMIC EDITOR:

- Visualization of obtained results must be improved

- Motivation behind proposed research should be more clearly explain. Please elaborate what is "beyond state-of-the-art" of proposed study.

- Make sure that the source code is available according to PLOS ONE publication policies.

- Make sure that you have conducted rigid statistical analysis.

**Answer:**

Dear editor:

1. Some of the figures and tables are redrawn and the captions are extended. Furthermore, some of the figures are deleted for briefly. For more details, please refer to the revised manuscript.

2. The sections of **Introduction** and **Related work** are revised thoroughly, some of the old references are removed and many recent works are added and analyzed. Please refer to the revised manuscript and the answers for the Reviewers for more details.

3. The source codes will be published on the github soon.

4. Two statistical analyses are conducted in the Experiment part, for more details please refer to the revised manuscript and the answers for the Reviewers.

## Reviewer #1

### Question 1:

*1. Include a more detailed comparative analysis with recent state-of-the-art methods in the field like below to highlight the advantages and limitations of the proposed approach.*

*Speech Emotion Recognition Using Deep Sparse Auto-Encoder Extreme Learning Machine with a New Weighting Scheme and Spectro-Temporal Features Along with Classical Feature …*

*Speech Emotion Recognition Using Multi-Layer Sparse Auto-Encoder Extreme Learning Machine and Spectral/Spectro-Temporal Features with New Weighting Method for Data Imbalance*

**Answer:**

In order to conduct more comprehensive analysis for the proposed method, the recent state-of-the-art methods mentioned above are included for a more detailed comparative analysis. The details of the modifications are listed below:

Section 2.3, 6^th^ paragraph,

Similarly, Fatemeh[27][28] proposed a hierarchical ELM (H-ELM) classification method, which consists of two successive stages, an unsupervised hierarchical feature representation and a supervised feature classification. In H-ELM, the weights of different classes were defined as follows:

$W_{ii}=\frac{1}{p_{i}+(Ny_{i}-p_{i})\times\frac{Ny_{i}}{\max(Ny_{i})}}$, $p_{i}=\sum_{i} Ny_{i}-Ny_{i}$

The corresponding references are added at the end of the manuscript,

[1] Daneshfar, Fatemeh, and Seyed Jahanshah Kabudian. "Speech Emotion Recognition Using Multi-Layer Sparse Auto-Encoder Extreme Learning Machine and Spectral/Spectro-Temporal Features with New Weighting Method for Data Imbalance." 2021 11th International Conference on Computer Engineering and Knowledge (ICCKE). IEEE, 2021.
[2] Daneshfar, Fatemeh, and Seyed Jahanshah Kabudian. "Speech Emotion Recognition Using Deep Sparse Auto-Encoder Extreme Learning Machine with a New Weighting Scheme and Spectro-Temporal Features Along with Classical Feature Selection and A New Quantum-Inspired Dimension Reduction Method." arxiv preprint arxiv:2111.07094 (2021).

These methods above are also added for comparison and the Experiments are extended, see the **Answer for Question 3, Reviewer #2** for more details.

Except for the 2 new references above, other 11 recent published articles are added for much deeper analyses, see the **Answer for Question 1, Reviewer #2** for more details.

### Question 2:

*2. Perform a sensitivity analysis on key parameters such as the number of reference points and the choice of kernel functions. This will help in understanding the robustness of the proposed model.*

**Answer:**

Dear professor, thank you for your suggestions.

In order to further explore the key parameters, a new section ‘5.2 Ablation Study’ is added in the 5^th^ section.

For the choice of reference points, our manuscript of the former vision has done enough experiment and analyses, as well as guidelines for the setting of reference point number. In the ablation study we add new analyses for the model selection and the choice of kernel function.

The details of the modifications are listed below:

**5.2	Ablation Study**

**To examine the effectiveness of each component in our proposed framework, a series of ablation experiments are performed on UCI dataset.**

**a) Evaluation on different hidden layer nodes and reference points**

**(please refer to the revised manuscript)**

**b) Evaluation on different models**

In this part, the effectiveness of the proposed two different models is investigated. For MKP-ELM and MKR-ELM, the kernel functions, No. of, NO. of $L_{1}$ are all the same. The cost matrix is randomly produced in a range of $[1,20]$. The average results on all datasets are presented in Table 2.

Table 2. comparison with different model

|  | Average $acc$(%) | | Average $Tc$ | |
| --- | --- | --- | --- | --- |
| Kernel function combination | MKP-ELM | MKR-ELM | MKP-ELM | MKR-ELM |
| $K_{1}(\cdot)$ | 83.22 | 83.26 | 9.44e1 | 9.43e1 |
| $K_{1}(\cdot)+K_{2}(\cdot)$ | 84.35 | 84.40 | 9.33e1 | 9.27e1 |
| $K_{1}(\cdot)+K_{2}(\cdot)+K_{3}(\cdot)$ | 84.34 | 84.41 | 9.36e1 | 9.27e1 |
| $K_{1}(\cdot)+K_{2}(\cdot)+K_{3}(\cdot)+K_{4}(\cdot)$ | 84.40 | 84.57 | 9.26e1 | 9.09e1 |

The results show that the performance of MKR-ELM is slightly better than that of MKP-ELM when there is only one kernel function, indicating that residual connection is effective for improving performance. When the kernel function is increased, the performance increase of MKP-ELM is smaller than that of MKR-ELM, indicating that firstly, increasing the kernel function can improve the performance, and secondly, vertically stacking multiple autoencoders with residual connections is more effective than simple horizontal expansion.

**c)** **Evaluation on different kernel combinations**

In this part, the effectiveness of different kernel function combination is investigated. In order to thoroughly analysis the influence of kernel function, we have exhausted all possible combinations under different number of kernel functions. For example, when the number of kernel functions is 2, we conduct six sets of experiments. The average results are shown in Table 3.

Table 3. Comparison with different kernel combination

|  | Average $acc$(%) | | Average $Tc$ | |
| --- | --- | --- | --- | --- |
| Kernel function combination | MKP-ELM | MKR-ELM | MKP-ELM | MKR-ELM |
| 1 kernel | 81.43 | 81.50 | 1.06e2 | 1.03e2 |
| 2 kernels | 83.77 | 83.98 | 9.88e1 | 9.80e1 |
| 3 kernels | 83.69 | 84.11 | 9.86e1 | 9.68e1 |
| 4 kernels | 84.40 | 84.57 | 9.26e1 | 9.09e1 |

The results in Table 2 and Table 3 both show that with the increase of the number of kernel functions, the performance of both models is improved. When the number of kernel functions is increased from 1 to 2, the performance increases significantly. Models with two and three kernel functions have similar performance, but when the number of kernel functions is increased from 3 to 4, the performance increases significantly. These indicate that different kernel functions have different feature mapping ability.

### Question 3:

3. *Discuss the potential real-world applications of the proposed method and how it can be integrated into existing systems. Providing a case study or example would strengthen the manuscript.*

**Answer:**

According to the suggestion, a case study on the realistic cost-sensitive problem is conducted. The experiments further verified the effectiveness of the proposed methods.

The details of the modifications are listed below:

**5.4 Case Study**

In this section, the proposed method is used for realistic cost-sensitive problems. LungHist700 is dataset of histological images in pulmonary pathology. It consists of 691 images from 45 patients, with each image having a resolution of 1200 × 1600 pixels and stored in .jpg format. These images are captured at either 20x or 40x magnification levels and are categorized into seven classes (see Figure 6). An accompanying.csv file links each image to the associated patient ID. All patients have been anonymized, and the file includes an identifier to match images from the same patient.

Figure 6. Example images of LungHist700

All the images are resized to 300*400 pixels, 70% for training and 30% for testing. A ResNet50 is trained and the last feature map is used as the input of MKR-ELM. For simplicity, the samples are divided into two classes, normal and unnormal. The misclassification costs of the normal and unnormal are 1 and 10, respectively. The results are presented in Table 7. ‘Normal’ indicates MKR-ELM without cost-sensitive classification. The results show that, cost-sensitive classification results in a 4.76% reduction in classification accuracy, but a 42.18% percent reduction in misclassification costs.

Table 7. Results on LungHist700

|  | Average $acc$(%) | | Average $Tc$ | |
| --- | --- | --- | --- | --- |
| methods | Normal | Cost-sensitive | Normal | Cost-sensitive |
| results | 81.56 | 76.8 | 1420 | 821 |

### Question 4:

*Minor Comments:*

*1. Expand the literature review to include more recent works related to cost-sensitive learning and multi-kernel ELMs.*

*2. Including pseudocode for the proposed algorithm could enhance understanding and reproducibility.*

*3. Ensure that all figures and tables are clearly labeled and referenced appropriately in the text. Some figures could benefit from more detailed captions.*

**Answer:**

1. In order to expand the literature review on cost-sensitive learning, the followings are added in 6^th^ paragraph, Section 1,

Cost-sensitive learning has drawn great attention in both theoretical researches and engineering practices. Theoretical researches can be divided into two categories, direct cost-sensitive learning and indirect cost-sensitive learning[23]. The former methods intended to use cost information to design new variants based on the basic classifiers, while the later ones treated the traditional classification algorithms as black boxes and performed preprocessing on the training data or post-processing on the outcomes with the cost information[24]. For engineering practices, cost-sensitive learning has been widely used in imbalanced classification[25], medical diagnosis[26], fault diagnosis[35], etc. Traditional ELM-based cost-sensitive learning mainly depended on the original ELM model[25]. Therefore, exploration based on other new variants of ELM is always needed.

The followings are added to further conclude existing works on multi-kernel ELM,

It has been demonstrated composite kernels could efficiently combines multi-level information and have excellent performance[17]. In [17], composite kernels is used for mapping contextual and spectral information,

$$K(\mathbf{x}_{i},\mathbf{x}_{j})=\mu K_{1}(\mathbf{x}_{i},\mathbf{x}_{j})+(1-\mu)K_{2}(\mathbf{x}_{i},\mathbf{x}_{j})$$

Where $\mu$is balance factor and $K_{1}(\mathbf{x}_{i},\mathbf{x}_{j})\text{，}K_{2}(\mathbf{x}_{i},\mathbf{x}_{j})$ are Radial basis function and polynomial function, respectively. The same methodology was adopted by [18]. However, the method above introduces one hyper-parameter, the balance factor, which is difficult to set. Therefore, [31] integrated multi-kernel matrix by adding each matrix immediately. Different from other articles that adopt traditional kernel functions, [26] redefined the kernel matrix as,

$$K(\mathbf{x}_{i},\mathbf{x}_{j})=\Phi_{1}(\mathbf{x})\cdot\Phi_{2}(\mathbf{x})\cdot\cdots\cdot\Phi_{n}(\mathbf{x})$$

Where $\Phi_{i}(\mathbf{x})$is the hidden layer output of a neural network. Instead of integrating multi-kernel functions into one super kernel, [23] proposed ML-KELM by stacking multiple KELM-AEs, which does not need to tune the parameters for all layers as in ML-ELM. In ML-KELM, each encoder has one distinct kernel function.

2. According to the comments, a pseudocode for the proposed algorithm is added as followings,

Algorithm 1. pseudocode for REKELM-AE

| Input |  |
| --- | --- |
| 1. | Randomly generate $\mathbf{W},\mathbf{b}$ according to $p(\mathbf{v})$ |
| 2. | Generate random mapping matrix $\mathbf{H}$ using $\mathbf{W},\mathbf{b}$ |
| 3. | Select  reference points from $\mathbf{H}$ randomly |
| 4. | Calculate the simplified matrix  according to Eq.(20) |
| 5. | Calculate output matrix $\mathbf{\Gamma}$according to Eq.(14) |
| Output | $\mathbf{\Gamma}$ |

3. According to the suggestions, some of the figures and tables are redrawn and the captions are extended. Furthermore, some of the figures are deleted for briefly. For more details, please refer to the revised manuscript.

## Reviewer #2:

### Question 1:

*1. Some of the references are very old, like [1], [7], I would recommend some of the following more recent papers dealing with swarm intelligence optimization of elm:*

*N. Bacanin, M. Zivkovic, M. Antonijevic, K. Venkatachalam, J. Lee, Y. Nam, M. Marjanovic, I. Strumberger, M. Abouhawwash, Addressing feature selection and extreme learning machine tuning by diversity-oriented social network search: an application for phishing websites detection, Complex & Intelligent Systems, pp. 1 - 36, Jun, 2023*

*Laifi, A., Benmohamed, E. & Ltifi, H. Xavier-PSO-ELM-based EEG signal classification method for predicting epileptic seizures. Multimed Tools Appl 83, 30675–30696 (2024). https://doi.org/10.1007/s11042-023-16514-3*

*Wang F, Liang Y, Lin Z, Zhou J, Zhou T. SSA-ELM: A Hybrid Learning Model for Short-Term Traffic Flow Forecasting. Mathematics. 2024; 12(12):1895. https://doi.org/10.3390/math12121895*

*N. Bacanin, C. Stoean, M. Zivkovic, D. Jovanovic, M. Antonijevic, D. Mladenovic, Multi-Swarm Algorithm for Extreme Learning Machine Optimization, SENSORS, Vol. 22, No. 11, pp. 1 - 34, May, 2022*

**Answer:**

According to the comments of all the reviewers, 10 of the original references, [1], [5],[6],[7], [8],[11],[15],[20],[22],[34], have been deleted. They are either too old or for briefly.

The recommended 4 recent papers dealing with swarm intelligence optimization of elm are added as References. Further, some other updated references that are also relevant to this article have been added. The details are listed as follows,

1. *The removed references,*

*~~[1]	G.-B. Huang, H. Zhou, X. Ding, et al. Extreme learning machine for regression and multiclass classification. IEEE Transactions on Systems, Man, ad Cybernetics-Part B: Cybernetics, 2012, 42(2): 513-529.~~*

*~~[5]	G.-B. Huang, L. Chen. Enhanced random search based incremental extreme learning machine. Neurocomputing, 2008, 71(16): 3460–3468.~~*

*~~[6]	Y. Miche, A. Sorjamma, P. Bas, et al. OP-ELM: Optimally pruned extreme learning machine. IEEE Transactions on Neural Networks, 2010, 21(1): 158-162.~~*

*~~[7]	Q.Y. Zhu, A.K. Qin, P.N. Suganthan, et al. Evolutionary extreme learning machine. Pattern Recognition, 2005, 38, 1759-1763.~~*

*~~[8]	Das S R, Mishra D, Lenka A, et al. Leveraging Cuckoo Search for Extreme Learning Machine for Mutual Fund Forecasting[C]//2024 International Conference on Emerging Systems and Intelligent Computing (ESIC). IEEE, 2024: 231-236.~~*

*~~[11]	P. Gastaldo, F. Bisio, S. Decherchi, et al. SIM-ELM: Connecting the ELM model with similarity-function learning. Neural Networks, 2016, 74: 22-34.~~*

*~~[15]	X. Liu, L. Wang, G.-B. Huang, et al. Multiple kernel extreme learning machine[J]. Neurocomputing, 2015, 149: 253-264.~~*

*~~[20]	N. Zhang, S. Ding, Z. Shi. Denoising Laplacian multi-layer extreme learning machine[J]. Neurocomputing, 2016, 171: 1066-1074.~~*

*~~[22]	T. Zou, Y. Luo, X.-H. Zhang, et al. Intelligent modeling using a novel feature extraction based multiple activation functions extreme learning machine[C]. Proceedings of the IEEE 16th International Conference on Networking, Sensing and Control, Alberta, Canana, May 9-11, 2019.~~*

*~~[34]	Z.-H. Zhou, X.-Y. Liu. Training cost-sensitive neural networks with methods addressing the class imbalance problem[J]. IEEE Transactions on Knowledge and Data Engineering, 2006, 18(1): 63-77.~~*

*2. The newly added references,*

*[1]	Rajput, Brajendra Singh, et al. "ELM-Based Imbalanced Data Classification-A Review." Informatica 48.2 (2024).*

*[5]	N. Bacanin, C. Stoean, M. Zivkovic, D. Jovanovic, M. Antonijevic, D. Mladenovic. Multi-Swarm Algorithm for Extreme Learning Machine Optimization, SENSORS, Vol. 22, No. 11, pp. 1 - 34, May, 2022.*

*[6]	N. Bacanin, M. Zivkovic, M. Antonijevic, K. Venkatachalam, J. Lee, Y. Nam, M. Marjanovic, I. Strumberger, M. Abouhawwash, Addressing feature selection and extreme learning machine tuning by diversity-oriented social network search: an application for phishing websites detection, Complex & Intelligent Systems, pp. 1 - 36, Jun, 2023.*

*[7]	Laifi, A., Benmohamed, E. & Ltifi, H. Xavier-PSO-ELM-based EEG signal classification method for predicting epileptic seizures. Multimed Tools Appl 83, 30675–30696 (2024).*

*[8]	Wang F, Liang Y, Lin Z, Zhou J, Zhou T. SSA-ELM: A Hybrid Learning Model for Short-Term Traffic Flow Forecasting. Mathematics. 2024; 12(12):1895*

*[19]	Zhang, Meng, et al. "An enhancing multiple kernel extreme learning machine based on deep learning." 2024 39th Youth Academic Annual Conference of Chinese Association of Automation (YAC). IEEE, 2024.*

*[20]	Zhao, Zhenchong, et al. "Cost-sensitive sample shifting in feature space." Pattern Analysis and Applications 23 (2020): 1689-1707.*

*[21]	Zhao, Zhenchong, and Xiaodan Wang. "Cost-sensitive SVDD models based on a sample selection approach." Applied Intelligence 48.11 (2018): 4247-4266.*

*[23]	Guan, Shan, et al. "A single-joint multi-task motor imagery EEG signal recognition method based on Empirical Wavelet and Multi‐Kernel Extreme Learning Machine." Journal of Neuroscience Methods 407 (2024): 110136.*

*[27]	Daneshfar, Fatemeh, and Seyed Jahanshah Kabudian. "Speech Emotion Recognition Using Multi-Layer Sparse Auto-Encoder Extreme Learning Machine and Spectral/Spectro-Temporal Features with New Weighting Method for Data Imbalance." 2021 11th International Conference on Computer Engineering and Knowledge (ICCKE). IEEE, 2021.*

*[28]	Daneshfar, Fatemeh, and Seyed Jahanshah Kabudian. "Speech Emotion Recognition Using Deep Sparse Auto-Encoder Extreme Learning Machine with a New Weighting Scheme and Spectro-Temporal Features Along with Classical Feature Selection and A New Quantum-Inspired Dimension Reduction Method." arxiv preprint arxiv:2111.07094 (2021).*

*[29]	Diosdado, J., Gilabert, P., Seguí, S. et al. LungHist700: A dataset of histological images for deep learning in pulmonary pathology. Sci Data 11, 1088 (2024).*

### Question 2:

*2. The author has used 60% of dataset for training and 40% for test set. This is not a common ratio. Can you elaborate on the reason for this exact split ratio?*

**Answer:**

**Dear professor:**

To our best knowledge, there is no universal criterion for the division of training data and test data. In [6], 80% of dataset for training and 40% for test set. In [5], 70% of data for training process, and 30% for testing. In [21], 10-fold cross validation was used while in [20] 66% for training and 34% for testing.

In our manuscript, the performance of different methods for cost-sensitive problems are compared. Using different amount of training data may influence the performance of each method. However, we think that, since all the methods used the same training and test dataset, they can be compared fairly.

As your suggestion, cross validation is adopted in the ongoing experiments. The results will be presented in the future vision.

### Question 3:

*3. I do not understand Table 2. Can you please clarify in the text (give an example) how to interpret the presented results in the Table 2?*

**Answer:**

Thank you for your suggestion.

In order to clearly present the experiments and further more interpret Table 2, the results of Type-a is presented as follows,

Every method runs 10 independent repetitions. Due to space limitations, only the results on Type-a is presented. Table 2 shows the average accuracy and misclassification cost. The “ ↓”,“ ↑” and “ ≈” marks imply that the result of MKP-CSELM is significantly better than, worse than and similar to the compared result with 0.05 significance level, respectively. In Table 2, in terms of accuracy, MKP-CSELM obtained the most optimal values (6), followed by mcKNN (4). As for the three data sets (Segmentation, Vehicle and Vowel), the accuracy of MKP-CSELM has been significantly improved compared with other methods, 3.0%, 3.3% and 7.3% higher than that of the second best method, and more than 20% higher than that of the worst method. For the misclassification cost, MKP-CSELM achieved optimal values on a total of 10 datasets, nearly half of the total number of datasets. Similarly, the misclassification cost of MKP-CSELM on Segmentation, Vehicle and Vowel data sets is significantly lower than that of other methods, indicating that the overall recognition performance of MKP-CSELM on the above three data sets has been greatly improved.

Further more, an example is given as follows, in order to interpret the presented results in the Table 2,

Each column indicates the number of dataset which MKP-CSELM is significantly better than, worse than and similar to the compared method with 0.05 significance level. For example, the first column under Type-a, 3,10,8 indicate MKP-CSELM is significantly worse than, similar to and better than CELM on 3,10,8 datasets, respectively.

## Reviewer #3:

### Question 1:

*1) A large number of formula definitions have been given in relevant work, but there are few literature summaries on the progress of* *ELM in cost-sensitive learning tasks, which needs to be further condensed;*

*2) Some formulas in the paper are ambiguous, and the characters of formula (19) are problematic;*

**Answer:**

According to the comments, some of the contents in section 2 are removed for briefly, the main modifications are as follows,

~~If a sample~~ ~~belongs to class~~~~, then the value of the~~~~-th element in~~ ~~is 1, and the rest are 0.~~

Let $\mathbf{h}(\mathbf{x})=[h_{1}(\mathbf{w}_{1},b_{1},\mathbf{x}),\cdots,h_{L}(\mathbf{w}_{L},b_{L},\mathbf{x})]$ ~~is the hidden layer output, which maps input samples~~ ~~from the~~~~-dimensional original feature space to the~~~~-dimensional hidden feature space~~, Eq. (1) can be rewritten in matrix form as:

$\mathbf{H\beta=T}$ (2)

~~Therefore, determining the output layer weights involves finding the solution to the linear system shown in Eq. (2). The solution for the initial output layer weights~~ ~~is obtained by minimizing the square error as follows using the method of minimum norm least squares:~~

 ~~(3)~~

~~The solution to the linear system can be represented as:~~

 ~~(4)~~

~~where~~ ~~is the Moore-Penrose pseudoinverse of~~~~.~~

~~In contrast to Eq. (3), the regularized ELM directly imposes the constraint on the norm of~~ ~~explicitly in the objective function. The optimal solution is obtained by solving the optimization problem with the following equality constraint:~~

 ~~(5)~~

~~where~~ ~~is the training error vector corresponding to the output layer nodes with respect to input~~~~, and~~ ~~is the regularization parameter.~~ The solution of Eq. (5) can be expressed in two forms:

$\left\{\begin{matrix} \mathbf{\beta}=\mathbf{H}^{T}(\frac{\mathbf{I}}{C}+\mathbf{HH}^{T})^{-1}\mathbf{T},\begin{matrix} \end{matrix}if:N<<L \\ \mathbf{\beta}=(\frac{\mathbf{I}}{C}+\mathbf{H}^{T}\mathbf{H})^{-1}\mathbf{H}^{T}\mathbf{T},\begin{matrix} \end{matrix}if:N>>L \end{matrix}\right.$ (6)

$C$ is the regularization parameter. When the form of $h_{j}(\cdot)$ is unknown, a kernel matrix can be defined for the ELM using the Mercer criterion:

$\mathbf{\Omega}=\mathbf{HH}^{T},\mathbf{\Omega}_{i,j}=h(\mathbf{x}_{i})\cdot h(\mathbf{x}_{j})=K(\mathbf{x}_{i},\mathbf{x}_{j})$ (7)

Replacing $\mathbf{HH}^{T}$ with $\mathbf{\Omega}$ in Eq. (7), the output function of KELM can be expressed as:

$f(\mathbf{x})=h(\mathbf{x})\mathbf{H}^{T}(\frac{\mathbf{I}}{C}+\mathbf{\Omega})^{-1}\mathbf{T}=\left[\begin{matrix} K(\mathbf{x}_{i},\mathbf{x}_{1}) \\ \begin{matrix} & \vdots\end{matrix} \\ K(\mathbf{x}_{i},\mathbf{x}_{N}) \end{matrix}\right](\frac{\mathbf{I}}{C}+\mathbf{\Omega})^{-1}\mathbf{T}$ (8)

ELM-AE [20] (as shown in Figure 1(a)) is essentially an ELM where the input and output are the same. ~~The learning process of ELM-AE is also a standard ELM training process. To distinguish it from ELM,~~ Let $\mathbf{\Gamma}$be the output weights of the autoencoder~~.~~

~~Replacing the output layer of Eq. (5) from~~ ~~to~~~~, the objective function of ELM-AE can be represented as:~~

 ~~(9)~~

The solution of ELM-AE in this case can be expressed as:

$\left\{\begin{matrix} \mathbf{\Gamma}=\mathbf{H}^{T}(\frac{\mathbf{I}}{C}+\mathbf{HH}^{T})^{-1}\mathbf{X},\begin{matrix} \end{matrix}if:N<<L \\ \mathbf{\Gamma}=(\frac{\mathbf{I}}{C}+\mathbf{H}^{T}\mathbf{H})^{-1}\mathbf{H}^{T}\mathbf{X},\begin{matrix} \end{matrix}if:N>>L \end{matrix}\right.$ (10)

~~Where~~ ~~is the feature matrix of input samples.~~ ~~After obtaining~~ ~~the abstract feature matrix~~ ~~obtained through autoencoder training can be expressed as:~~

 (11)

~~To obtain more sparse and compact features, Tang[22] used the L1 norm of the output weights instead of the L2 norm in Eq. (9). In this case, the objective function of ELM sparse autoencoder can be expressed as:~~

 ~~(12)~~

The reviews on ELM in cost-sensitive learning tasks have been extended. For more details, please refer to the **Answers** for **Question1** and **Question 4, Reviewer #1**.

Dear professor,

With regard to formula (19) (Eq.(16) in the revised manuscripe), the output $\mathbf{\Gamma}$ is a matrix. Put $\mathbf{\Gamma}$ in formula (18), the result is correct. Furthermore, formula (19) is derived by a strict formula reasoning according to the related reference. We are confused about this comment. We will appreciate it if you can give more comments about this problem.

### Question 2:

*3) In the fourth part of the manuscript, the feature mapping is carried out by combining multiple ELM. The expression of how the output k feature values correspond to m hidden layer vectors is not clear and lacks innovation;*

**Answer:**

Dear professor,

The way how the output k feature values correspond to m hidden layer is presented in the manuscript after Eq.(25), as follows,

‘After obtaining $k$ abstract features extracted by REKELM-AEs, combine all the results into a feature matrix $\mathbf{X}^{final}=[\mathbf{X}^{(1)},\mathbf{X}^{(2)},\cdots,\mathbf{X}^{(k)}]$, and finally use $\mathbf{X}^{final}$ as input to train an ELM classifier $\mathbf{H}^{final}\mathbf{\beta}=\mathbf{T}$’

In order to further extend the innovation of the manuscript, the proposed models are extended to 2. The modification are presented as follows,

**4. Cost-sensitive ELM with Multi-kernel Autoencoder**

This section first proposes two classification models based on ~~multi-kernel parallel ELM (MKP-ELM) according to~~ REKELM-AE, namely multi-kernel parallel ELM (MKP-ELM) and multi-kernel residual ELM (MKR-ELM). Then, the cost-sensitivity models ~~of MKP-ELM is~~ are realized according to the minimum risk decision theory.

4.1 MKP-ELM and MKR-ELM

Most existing AE-based neural network models achieve deep representation of inputs by vertically stacking multiple AEs. However, this layer-by-layer learning strategy accumulates reconstruction errors in the process of feature reconstruction, leading to the learned features cannot represent the original input information well [23]. On the other hand, different kernel functions have different expressive capabilities for different data. By combining multiple kernel functions for learning, we can leverage the advantages of different kernel functions, obtaining better flexibility and adaptability. Based on the REKELM-AE proposed in the third section, this paper presents two multi-kernel autoencoder-based ELM models, multi-kernel parallel ELM (MKP-ELM) and multi-kernel residual ELM (MKR-ELM), as shown in Figure 3.

Figure 3: Network Structure of MKP-ELM and MKR-ELM.

Residual connection has been proved to be an effective and efficient principle in deep neural networks[2][3]. In order to further exploit the effectiveness of the proposed RKELM-AE, another deep model is designed by stacking multiple REKELM-AEs. Furthermore, the residual connection is adopted between each autoencoder, as illustrated in Figure 3. The output $\mathbf{Y}^{(i)}$of one REKELM-AE is,

$\mathbf{Y}^{(i)}=\mathbf{X}^{input}+\mathbf{X}^{output}$ (26)

Where $\mathbf{X}^{input},\mathbf{X}^{output}$ are the input and output of the encoder. Different REKELM-AE has different kernel function.

Stacking multiple REKELM-AEs can obtain multiple kernel mappings, while residual connection eases the information loss during the transmission.

### Question 3:

4) Detailed introduction of baseline methods and ablation experiments were lacking in comparison experiments

**Answer:**

In order to further explore the key parameters, a new section ‘5.2 Ablation Study’ is added in the 5^th^ section. Please refer to the **Answers** for **Question2, Reviewer #1** for more details.
